# Supplementary material for: CEP290 deficiency disrupts ciliary axonemal architecture in human iPSC-derived brain organoids
Source: J Cell Sci. 2025 Oct 31;138(20):jcs264092. doi: 10.1242/jcs.264092 (PMC12633733; doi:10.1242/jcs.264092)
Supplement: Supplementary information [file joces-138-264092-s1.pdf]

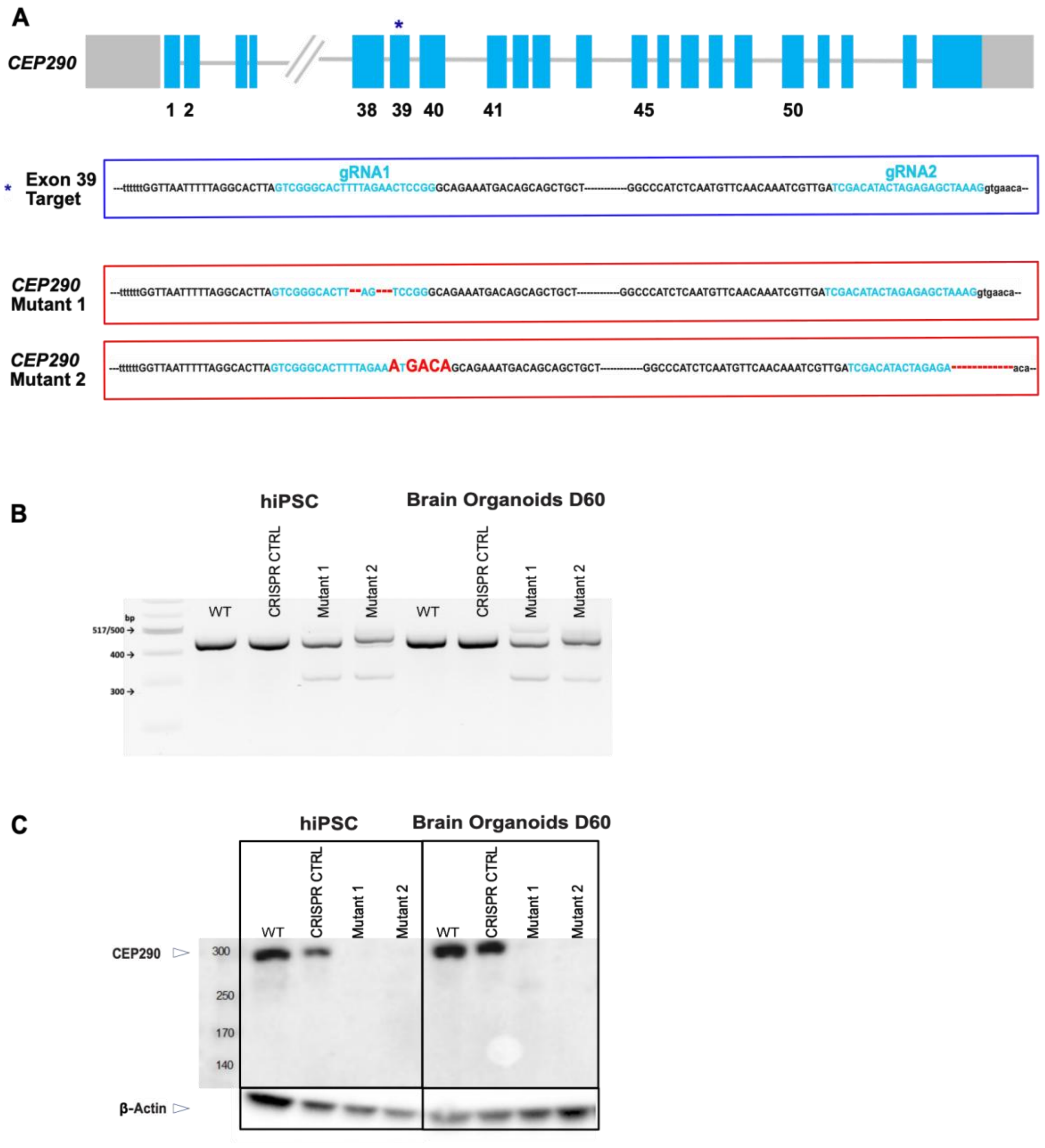

Fig. S1. *CEP290* mutation effect in hiPSC and brain organoids

(A) Schematic diagram showing the locations of the two sgRNAs target sites in the *CEP290* gene used to CRISPR-edit the control hiPSC line ISFi001-A clone 5 monoclonal line. The star marks exon 39 which was targeted. In the reference sequence (blue box), lower case indicates intronic sequence. Red boxes show the *CEP290* mutations introduced at the genomic level. **(B)** Agarose gel of endpoint RT-

Journal of Cell Science • Supplementary information

PCR in control (wildtype WT) and *CEP290* mutant hiPSCs (left) and 60-day old brain organoids (right). CRISPR CTRL is a line that underwent CRIPSR'ing but did not result in an edit at the target sites. *CEP290* Mutant 1 and 2 show reduced full-size bands (Sanger sequencing demonstrated that these bands include the small indels indicated in (A)) and an additional shorter band representing mRNA with skipped exon 39. This pattern is identical in iPSCs and in 60-day cultured brain organoids. **(C)** Western blot of CEP290 in *CEP290* mutants and control hiPSC (left) and 60-day old brain organoids (right). Loading control is shown below (beta-actin). Upon loading similar amount (10μg) of protein, we did not detect any CEP290 bands in mutants at hiPSC or 60-day old brain organoid level. When loading higher protein amounts (30μg), a very faint band becomes apparent likely representing minimal amounts of residual CEP290 protein lacking exon 39 (shown in Figueiro-Silva et al, https://doi.10.1016/j.scr.2025.103781)

Journal of Cell Science • Supplementary information

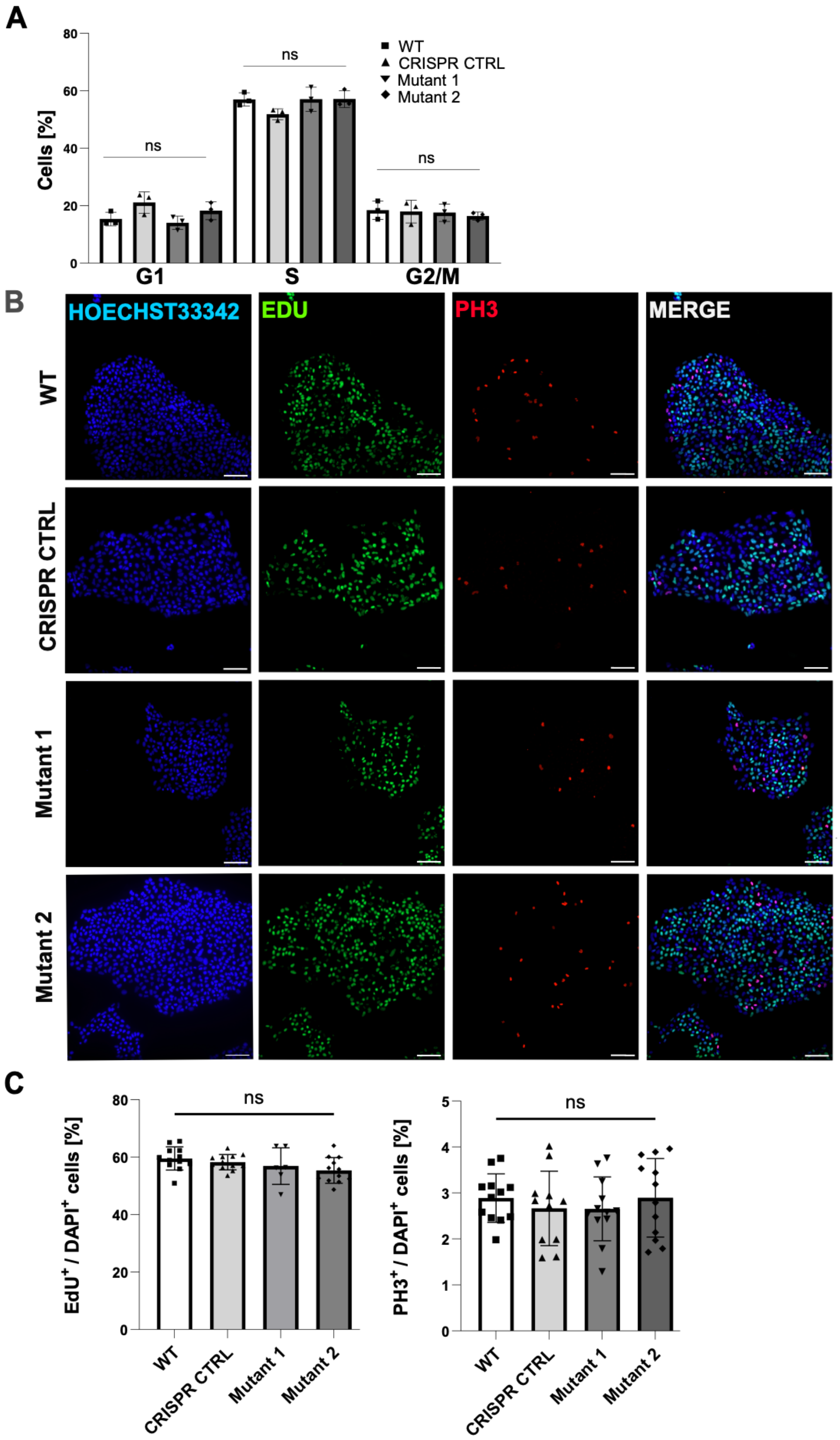

Fig. S2. Loss of CEP290 does not affect cell cycle in hiPSCs

(A) Bar graph showing quantification of the relative percentage of cells in cell cycle phases G1, S and G2/M of hiPSC control (WT and CRISPR control) and *CEP290* mutant (Mutant 1 (p. L1749Sfs*10) and Mutant 2 (p. L1787Hfs*12) by flow cytometry. Error bars show mean±SD. One-way Anova with Dunnett's multiple comparison test: ns>0.05, n= 3 biological replicates. **(B)** Widefield images of control (WT and CRISPR control) and *CEP290* mutants (Mutant 1 and 2) iPSC colonies stained by immunocytochemistry for cell cycle markers EdU (green) and PH3 (red). Nuclei were

Journal of Cell Science • Supplementary information

stained with DAPI(blue). Scale bars: 100 µm. **(C)** Quantification of EdU /DAPI and PH3DAPIcells on immunocytochemistry reveals no significant difference between control and mutant *CEP290* hiPSC lines. Error bars show ±SD. One-way Anova with Dunnett’s multiple comparison test: ns>0.05, n=3 biological replicates, for each 6 image fields were analysed.

Journal of Cell Science • Supplementary information

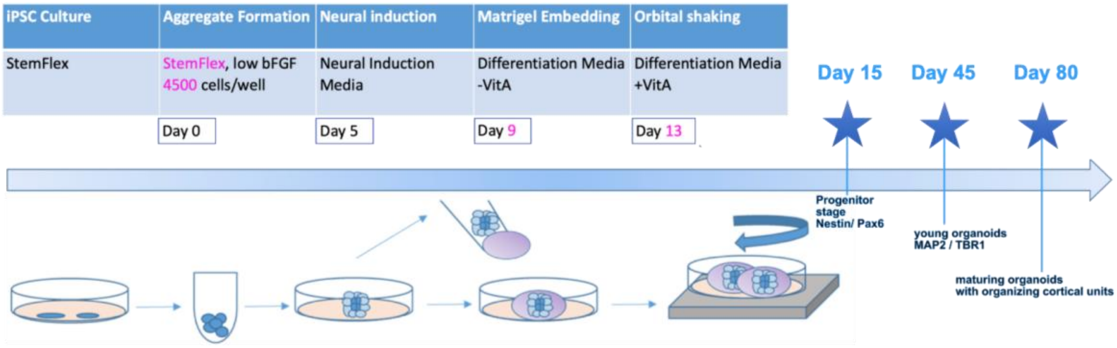

Fig. S3. Optimized workflow for generating brain organoids from hiPSCs and overview of analysis timepoints

hiPSCs are maintained in StemFlex medium™ (SF). The schematic overview shows the optimized workflow to generate brain organoids from the HMGU#1 clone 5 and derived CRISPR control and two CEP290 mutant hiPSC lines. Changes from the original protocol described in Lancaster et al 2014 (https://doi.org/10.1038/nprot.2014.158) have been highlighted in pink. 4500 hiPSCs are seeded/well in one full 96well ultra-low attachment plate at Day 0, cultured in SF medium with additionally supplementing low basic-fibroblast-growth factor (bFGF) and Rock-Inhibitor (RI). Day 5: neural induction; Day 9: Matrigel embedding followed by 4 days of stationary culture in differentiation medium without Vitamin A. Subsequent long-term culture and differentiation of the organoids up to 80 days on an orbital shaker in differentiation medium containing Vitamin A. Downstream endpoint analyses were performed on day 15 of culture at the progenitor stage (cultures should express Nestin and Pax6), at day 45 of culture by validating presence of neuronal marker MAP2 and post-mitotic neuronal marker TBR1 and final endpoint at day 80 in which brain organoids are more mature by exhibiting the start of organizing cortical units.

Journal of Cell Science • Supplementary information

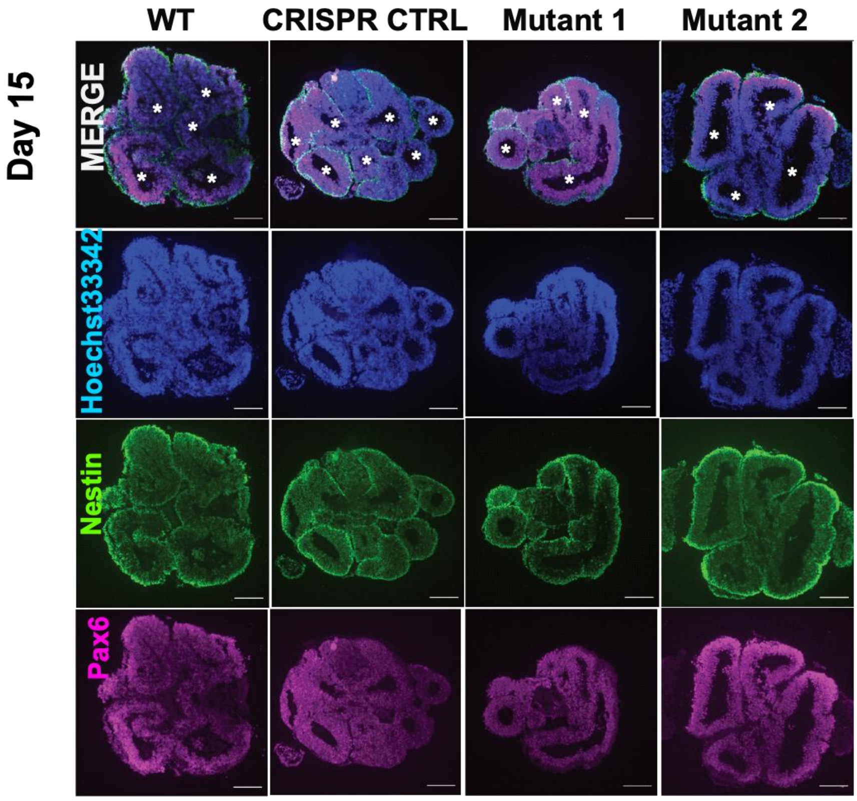

Fig. S4. Brain organoids at day 15 exhibit similar progenitor marker expression and organization in *CEP290* mutant and control cultures Widefield images of cryosectioned control and *CEP290* mutant brain organoids at day 15. Immunohistochemistry of neural progenitor marker Nestin (green), neural progenitor marker PAX6 (purple) and nuclear counterstain Hoechst33342. *CEP290* mutant and control cells build comparable organoid morphologies at this stage. This figure shows separate channels for the overview widefield images shown in Fig. 1B. Ventricular lumen are marked by asterisks. Scale bars are 200 µm.

Journal of Cell Science • Supplementary information

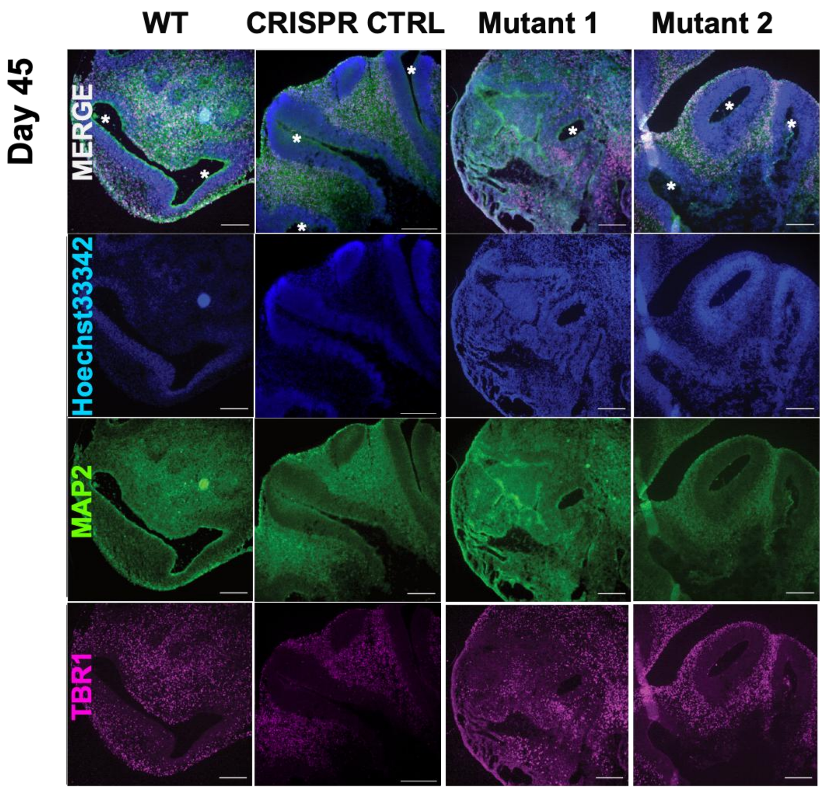

Fig. S5. 45-day old *CEP290* mutant and control brain organoids express post-mitotic neural markers MAP2 and TBR1

Widefield images of cryosectioned control and *CEP290* mutant brain organoids at day 45. Immunohistochemistry showing mature neuronal marker MAP2a/b (green) and post-mitotic neuronal marker TBR1 (purple). *CEP290* mutants and controls express both mature neuronal marker MAP2a/b and post-mitotic neural marker TBR1, which are located outside the ventricular zones. *CEP290* mutant 1 shows more disorganised and villous-like structures compared to controls and to *CEP290* Mutant 2. Nuclear counterstaining with Hoechst33342 (blue). This figure shows separate channels for the overview widefield images shown in Fig. 1C. Ventricular lumen are marked by asterisks. Scale bars: 200 μm.

Journal of Cell Science • Supplementary information

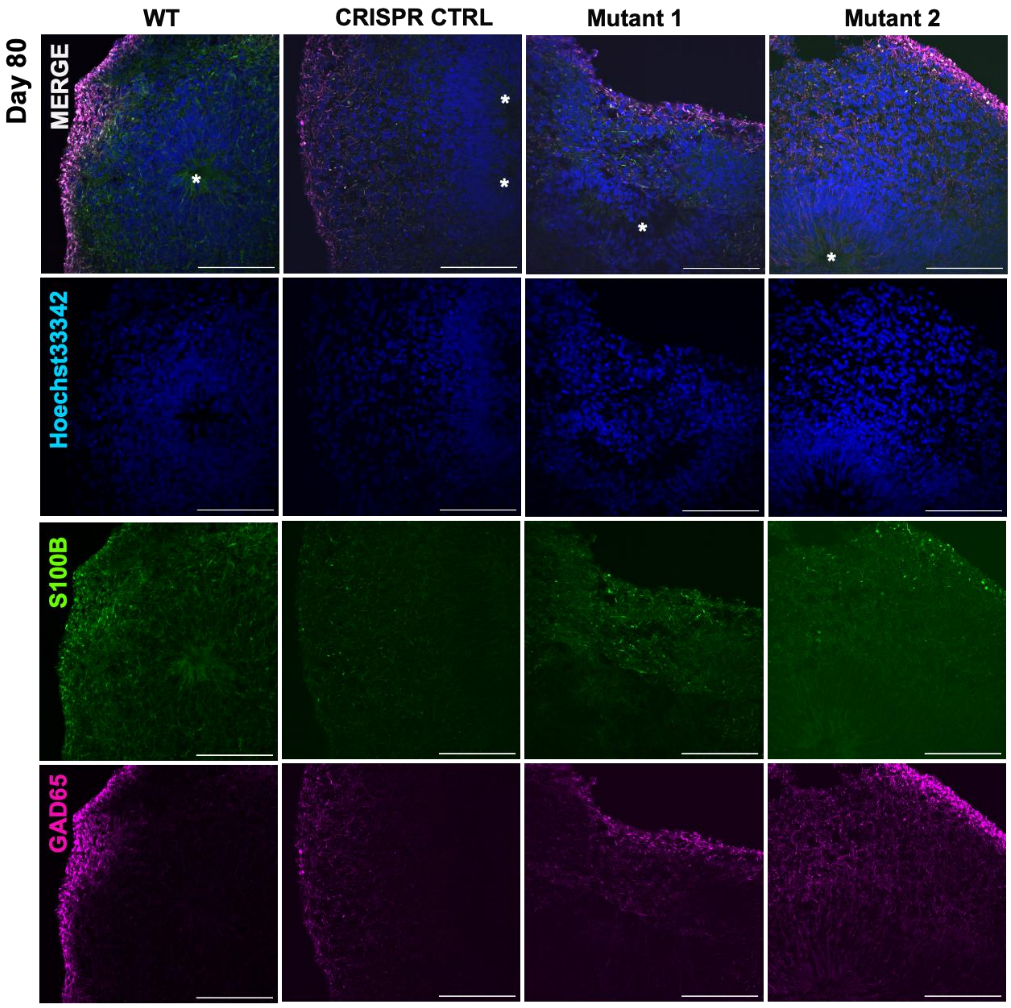

Fig. S6. 80-day-old *CEP290* mutant and control organoids produce glial cells and GABAergic interneurons

Confocal immunohistochemistry images of cryosections of 80-day old cerebral organoids stained with glial cell marker S100B (green) and GABAergic interneuron marker GAD65 (purple), with nuclear counterstain Hoechst33342 (blue). *CEP290* mutants and controls all show positive staining for S100B and GAD65 with similar organization. Ventricular lumen are marked by asterisks. Scale bars: 100 μm.

Journal of Cell Science • Supplementary information

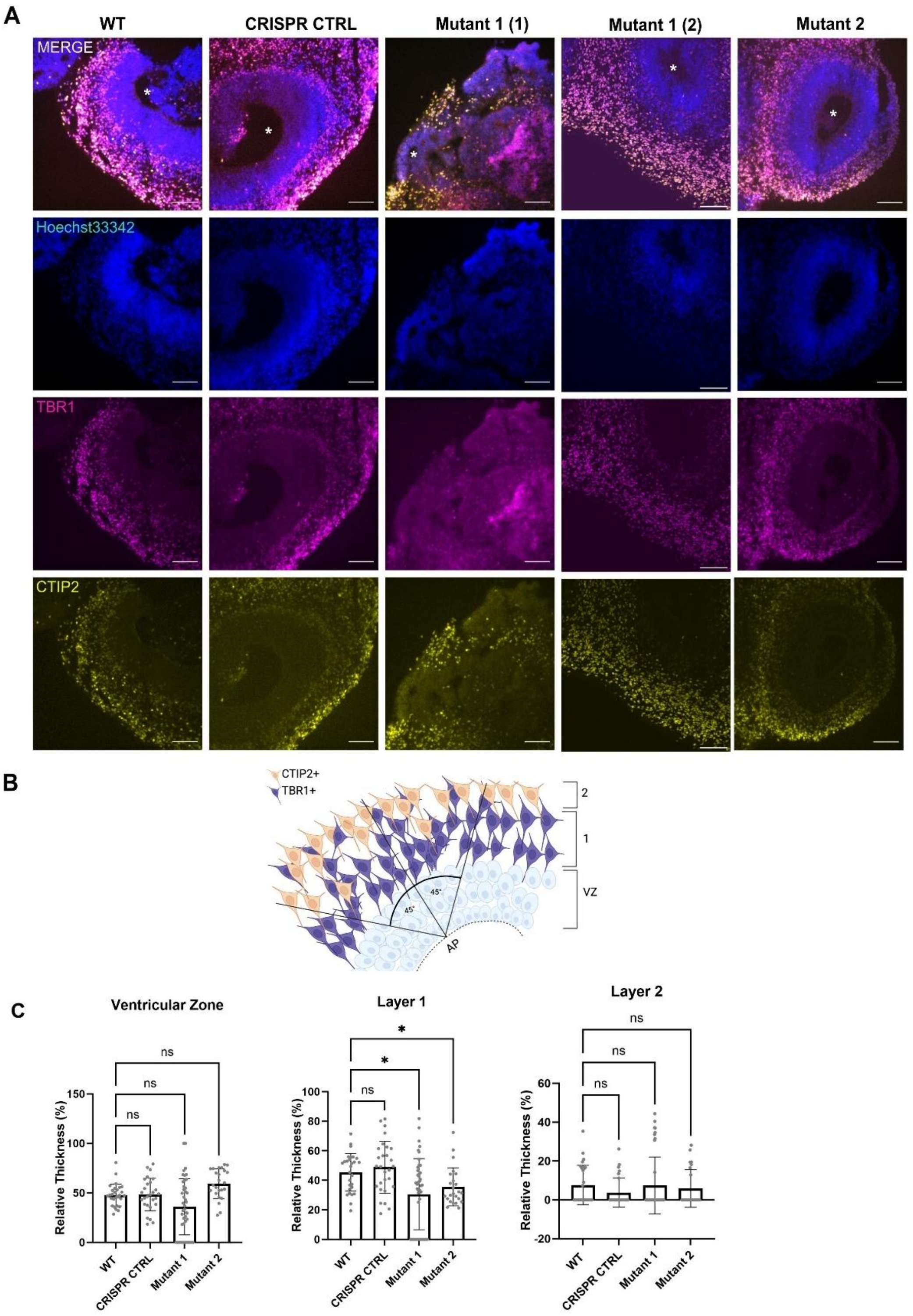

Fig. S7. Cortical layer formation in *CEP290* mutant brain organoids

(A) Representative widefield immunohistochemistry images of cryosections of 80-day old brain organoids stained with post-mitotic neuronal marker TBR1 and layer V/VI marker CTIP2 (yellow), counterstained with nuclear stain Hoechst33342 (blue).

Journal of Cell Science • Supplementary information

CEP290 mutants and controls all show positive staining for TBR1 and CTIP2, but organization of the layers is occasionally disrupted in organoids generated from Mutant 1 (two representative examples of the appearance of Mutant 1organoids are shown: disrupted organization (Mutant 1 (1)); intact cortical unit organization (Mutant 1 (2)). However, this was highly variable and not reproduced in all differentiation runs. Such disorganized was not observed in Mutant 2. Ventricular lumen are marked by asterisks. Scale bars: 200 µm. **(B)** Schematic illustration for quantifying cortical layering in brain organoids: ventricular zone (VZ, blue), Layer 1 (purple, mostly $TBR1^+$ cells) and layer 2 (mixed population of $TBR1^+$ and $CTIP2^+$ cells). Relative thickness of these layers was measured and averaged along three lines 45° angles apart as indicated. **(C)** Quantitative analysis of relative thickness of VZ, Layer 1 and Layer 2 in 80-day old control and *CEP290* mutant brain organoids. *CEP290* mutants shows slightly significantly decreased layer 1 thickness. Error bars show ±SD. Kruskal Wallis multiple comparison test: ns>0.05, *p<0.05. Data was obtained from three independent differentiations (N=3), for each differentiation three cortical structures from three organoids were assessed.

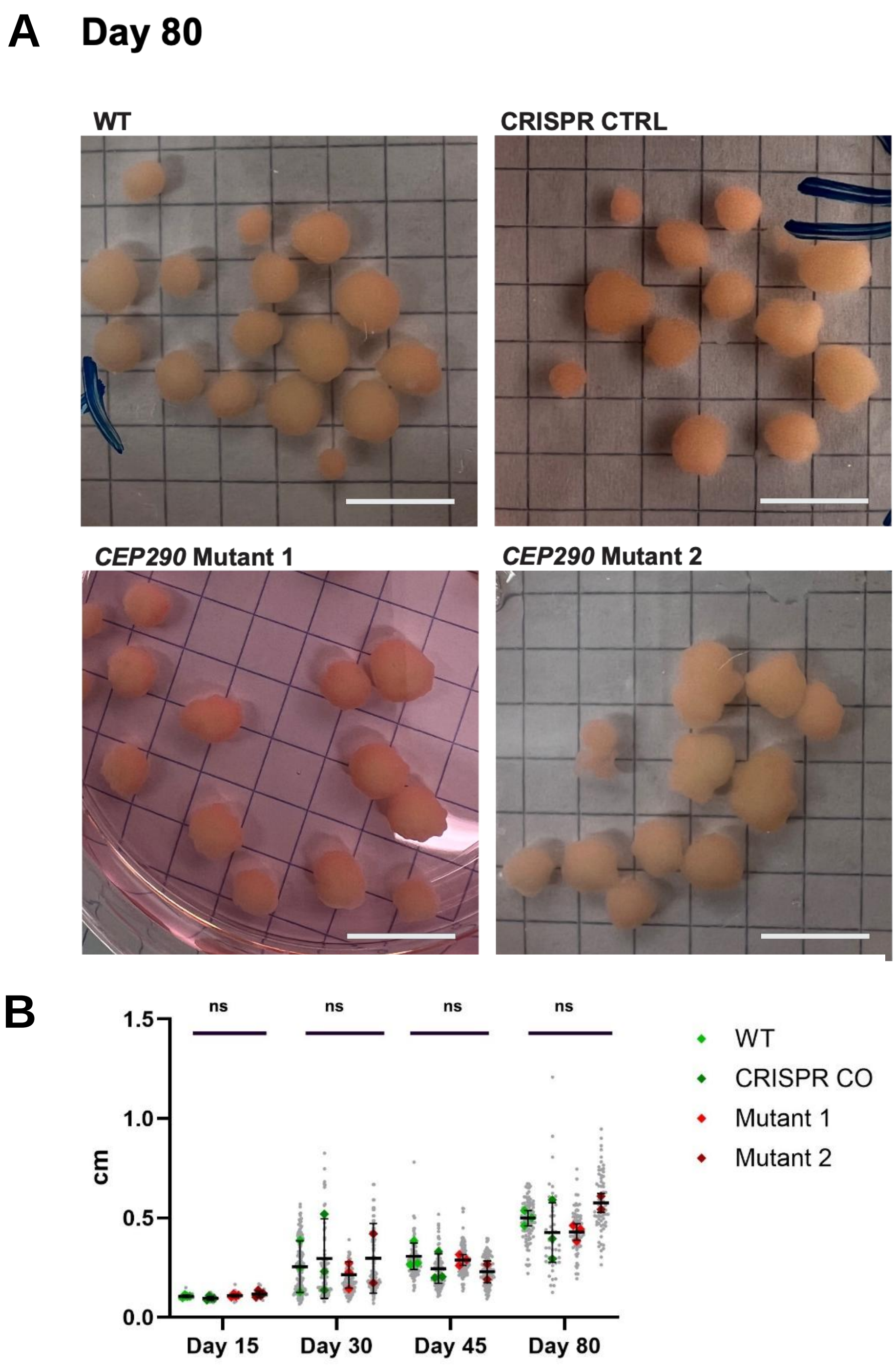

Fig. S8. Growth of mutant organoids is not significantly different compares to controls

(A) Overview images of multiple organoids in wells of wildtype, CRISPR control and both mutant cultures at day 80. Scale bars: 1cm. Note that the variability in organoid size within each well is expected with this protocol. **(B)** Average diameter of organoids at days 15, 30, 45 and 80 in controls and *CEP290* mutant organoids. Graph shows mean ±SD in color and in grey measurements of individual organoids (note the wide spread in organoid sizes in all conditions starting at day 30), 2 way-Anova performed on averages and Tukey's multiple comparison test: ns $p>0.05$ (3-4 independent differentiations (N=3-4)).

Journal of Cell Science • Supplementary information

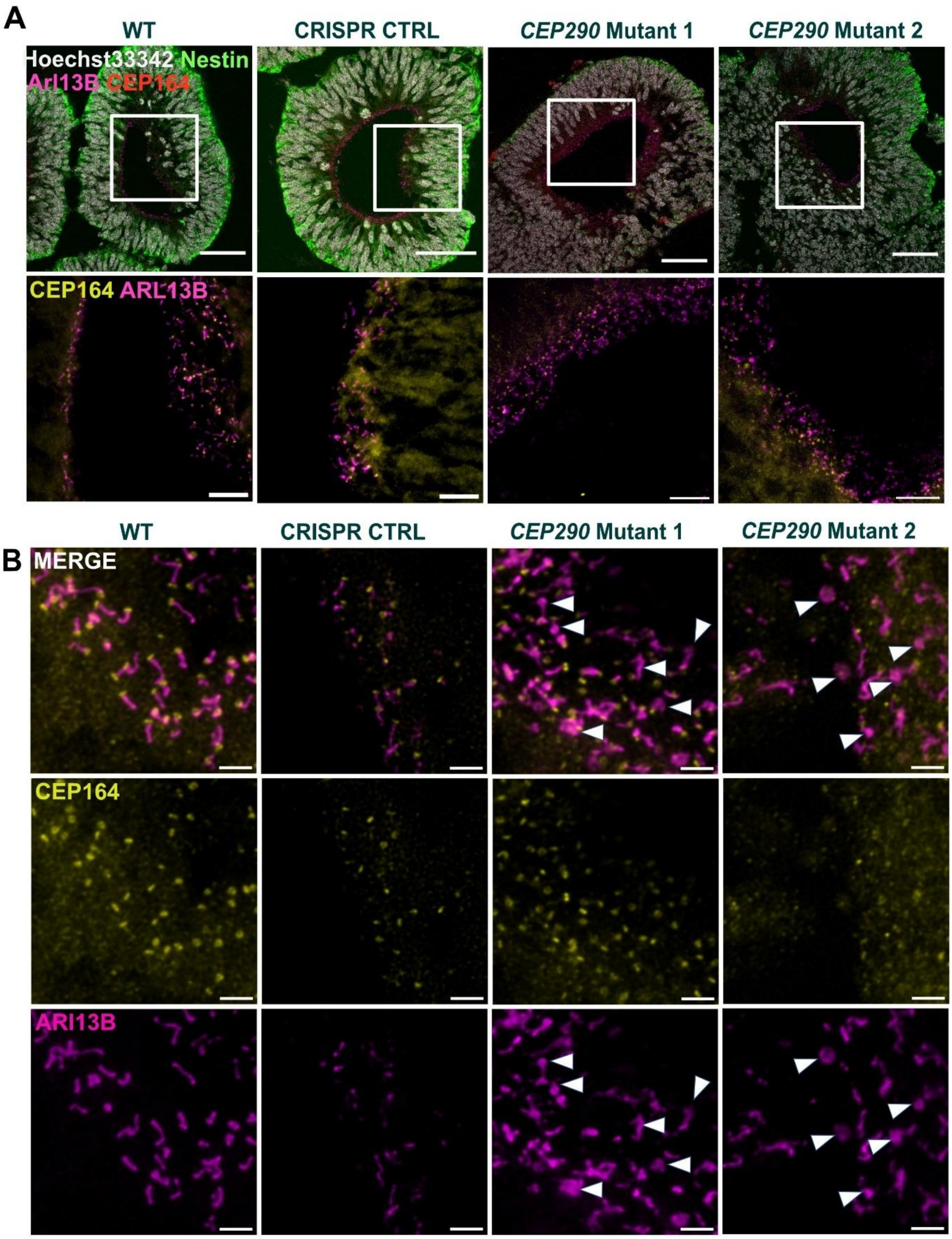

Fig. S9. Overview immunofluorescence images of ventricular lumen cilia in *CEP290* mutant and control brain organoids

(A) Confocal images of immunohistochemistry from 15-day old brain organoids. The top panel shows staining for the neural stem cell marker Nestin (green), ciliary

Journal of Cell Science • Supplementary information

membrane marker ARL13B (purple), ciliary base marker CEP164 (yellow) and nuclear counterstain Hoechst33342 (white). Cilia project from the apical side of neural stem cells into the ventricular lumen in both controls and *CEP290* mutant brain organoids. The lower panel shows magnified regions within the ventricular lumen (boxed in the top panel). Scale bars are 20 µm (upper panel) and 10 µm (lower panel). **(B)** Confocal images of immunohistochemistry on cryosections from 30-day old brain organoids, stained for the ciliary membrane marker ARL13B (purple) and basal body marker CEP164 (yellow). Both control and *CEP290* mutant organoids exhibit comparable ciliation within ventricular lumen, with expression of ARL13B and CEP164. A subset of cilia in mutant brain organoids, but rarely in controls, display morphological abnormalities (arrowheads). Scale bars: 5 µm.

Journal of Cell Science • Supplementary information

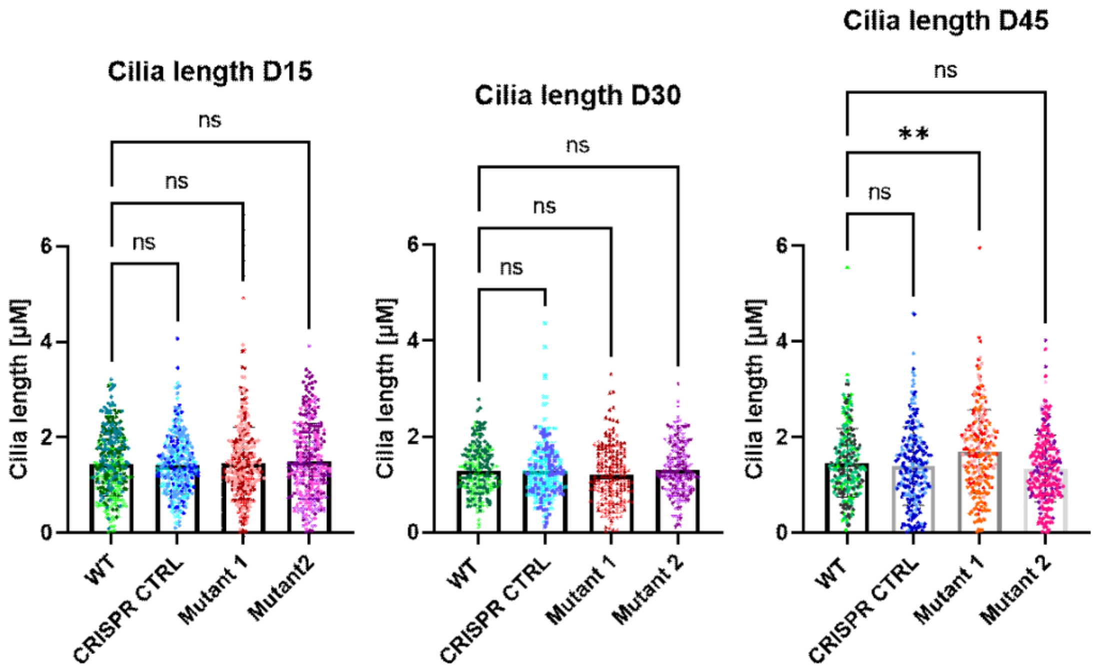

Fig. S10. Length of cilia in ventricular lumen is largely unaffected in *CEP290* mutant brain organoids at day 15, 30 and 45 of differentiation

Each dot represents the measurement of one single cilium and the different color shades for each scatterplot represent cilia measurements for each independent organoid differentiations. Length quantification was performed with ImageJ/Fiji plugin CiliaQ of deconvoluted images. Only cilia with the typical rod-shape were measured. Cilia images were obtained at ventricular surfaces with a SP8 confocal microscope at 63x magnification and a 7.5x zoom with consistent laser settings. Cilia from 3 ventricular lumen of 3 separate organoids were analyzed, from each of 3 independent experiments (n=9 biological replicates). Statistical analysis was performed with Kruskal Wallis multiple comparison test: ns>0.05, **p<0.01.

Journal of Cell Science • Supplementary information

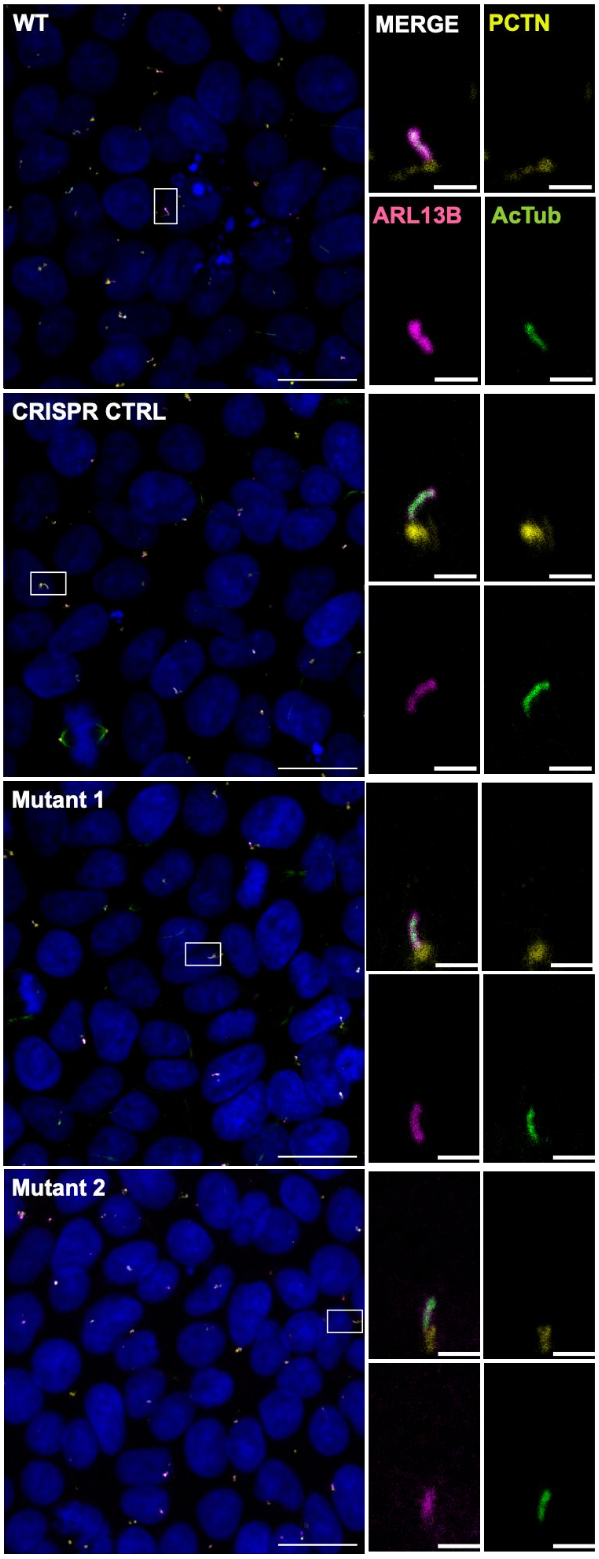

Journal of Cell Science • Supplementary information

Fig. S11. Cilia of *CEP290* mutant hiPSCs are indistinguishable from control

Confocal images of immunohistochemistry from control and *CEP290* mutant hiPSC cultures showing cilia, stained for the ciliary membrane marker ARL13b (purple), Acetylated tubulin (green), ciliary base marker pericentrin (yellow) and nuclear counterstain Hoechst33342 (blue). The left panels show an overview of the culture (scale bars are 20 μm) showing similar ciliation rates and the right panels show magnified cilia (boxed in the left panel), that appear morphologically normal (scale bars are 5 μm).

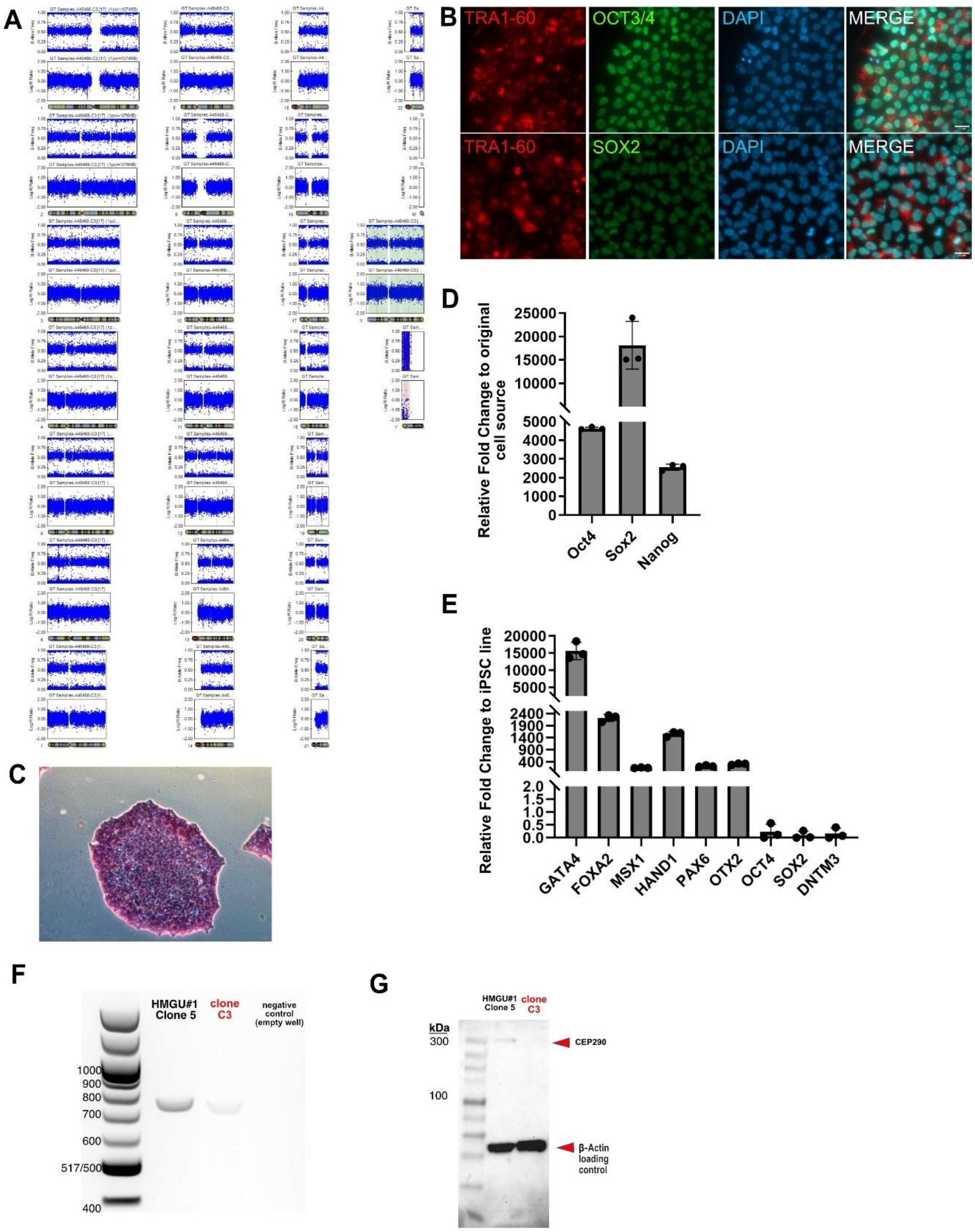

Fig. S12. Quality control of reprogrammed patient-hiPSC with *CEP290* mutations

(A) Copy-number variant (CNV) analysis of reprogrammed patient-hiPSC line with Array-comparative genomic hybridization (aCGH). **(B)** The selected patient-derived hiPSC clone C3 is positive on immunolabeling for marker Oct3 (green), Tra-1-60 (red)

Journal of Cell Science • Supplementary information

and DAPI (blue) (upper panel) as well as SOX2 (green) (lower panel). Scale bars are 100 µm. **(C)** Alkaline phosphatase staining in patient-hiPSC Clone C3. Scalebar: 200 µm. **(D)** Quality control qRT-PCR for pluripotency markers Oct4, Sox2 and Nanog in the selected clone C3. Data are normalized to GAPDH and relative to the original cell source (amniocytes). Quantitative data are presented as mean ± standard deviation (n = 3). **(E)** Quality control qRT-PCR for differentiation potential into the three germlayers for the selected clone C3. Data are normalized to GAPDH and relative to undifferentiated hiPSC form clone C3. Quantitative data are presented as mean ± standard deviation (n = 3). **(F)** Endpoint RT-PCR agarose gel showing that the patient-derived selected hiPSC Clone C3 has strongly reduced *CEP290* cDNA compared to WT control hiPSC Clone 5. **(G)** CEP290 Western Blot of reprogrammed patient-derived C3 clone hiPSC and control HMGU1 clone 5: Upon loading (10 µg) of protein, we did not detect any bands against CEP290 in patient-derived *CEP290* hiPSC protein lysates.

Journal of Cell Science • Supplementary information

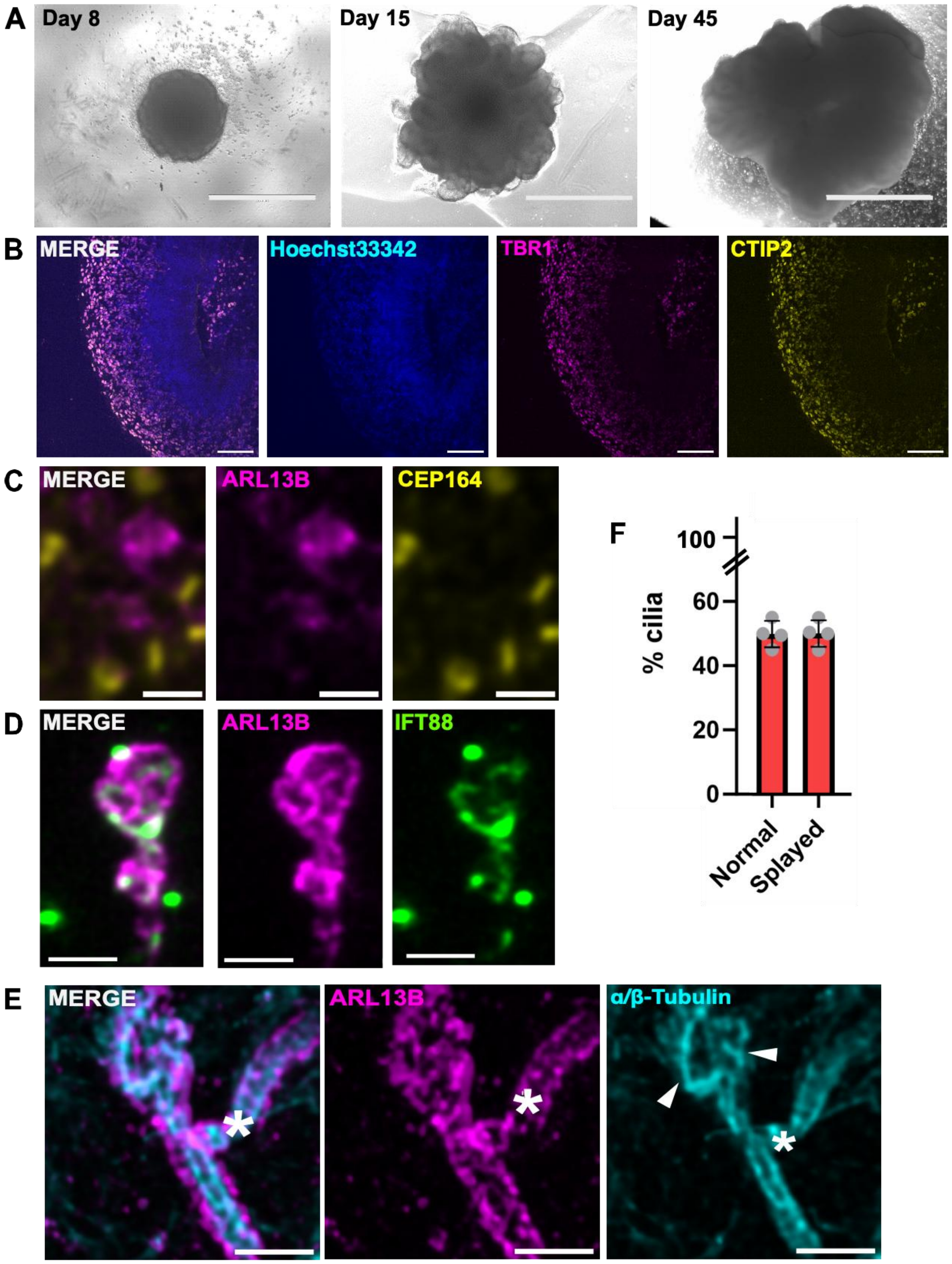

Fig. S13. ***CEP290*** **patient-derived hiPSC generate brain organoids with ciliary abnormalities**

(A) Brightfield images of brain organoids generated from *CEP290* patient-derived hiPSCs (hiPSCs reprogrammed from amniocytes carrying biallelic *CEP290* truncating variants (homozygous c.5493del, p.(Ala1832Profs*19). Patient-derived brain

Journal of Cell Science • Supplementary information

organoids show compact 3D aggregates with neural induction (brighter surface) at day 8 (marked by arrowhead), visible budding after Matrigel embedding at day 15 and prominent lumen of cortical units at day 45 of differentiation (marked by arrowheads). Scalebars day 8 and 15: 1000 µm; day 45: 2000 µm. **(B)** Widefield immunohistochemistry image of a cryosection of 80-day old patient-hiPSC derived brain organoid stained with TBR1 (purple) and CTIP2 (yellow), counterstained with nuclear stain Hoechst33342 (blue). Patient-hiPSC derived brain organoids show positive staining for both markers. Ventricular lumen marked by asterisk. Scale bars: 200 µm. **(C)** Immunohistochemistry 45-day-old brain organoids focusing on cilia in the ventricular lumen stained with anti-CEP164 (basal body marker) in yellow and anti- ARL13B (ciliary membrane marker) in purple, scale bars: 2 µm. Note the racket-like shape of the cilium. **(D)** Immunohistochemistry with expansion super-resolution microscopy (UxEM) of cryosectioned 30-day old patient-hiSPC derived organoid with CEP290 mutations stained with ciliary membrane marker ARL13B (purple) and IFT88 (green). Scale bars (post-expansion physical scale): 2.1 µm (corresponds to 500 nm, corrected for 4.2 expansion factor). **(E)** UxEM of cryosectioned 30-day old patient-hiPSC derived brain organoid stained with ciliary marker ARL13B (purple) and axonemal microtubule marker α/β-tubulin (cyan), showing two cilia. The arrowheads point to splayed microtubules in one expanded ciliary tip, while the asterisk indicates a second cilium nearby. Scale bars (post-expansion physical scale): 2.1 µm (corresponds to 500 nm, corrected for 4.2 expansion factor). **(F)** Quantification of normal and abnormal (splayed microtubule staining) cilia of overview images in UxEM. Graph shows mean ±SD (each datapoint represents the frequency of splayed microtubules in one biological replicate).

Journal of Cell Science • Supplementary information

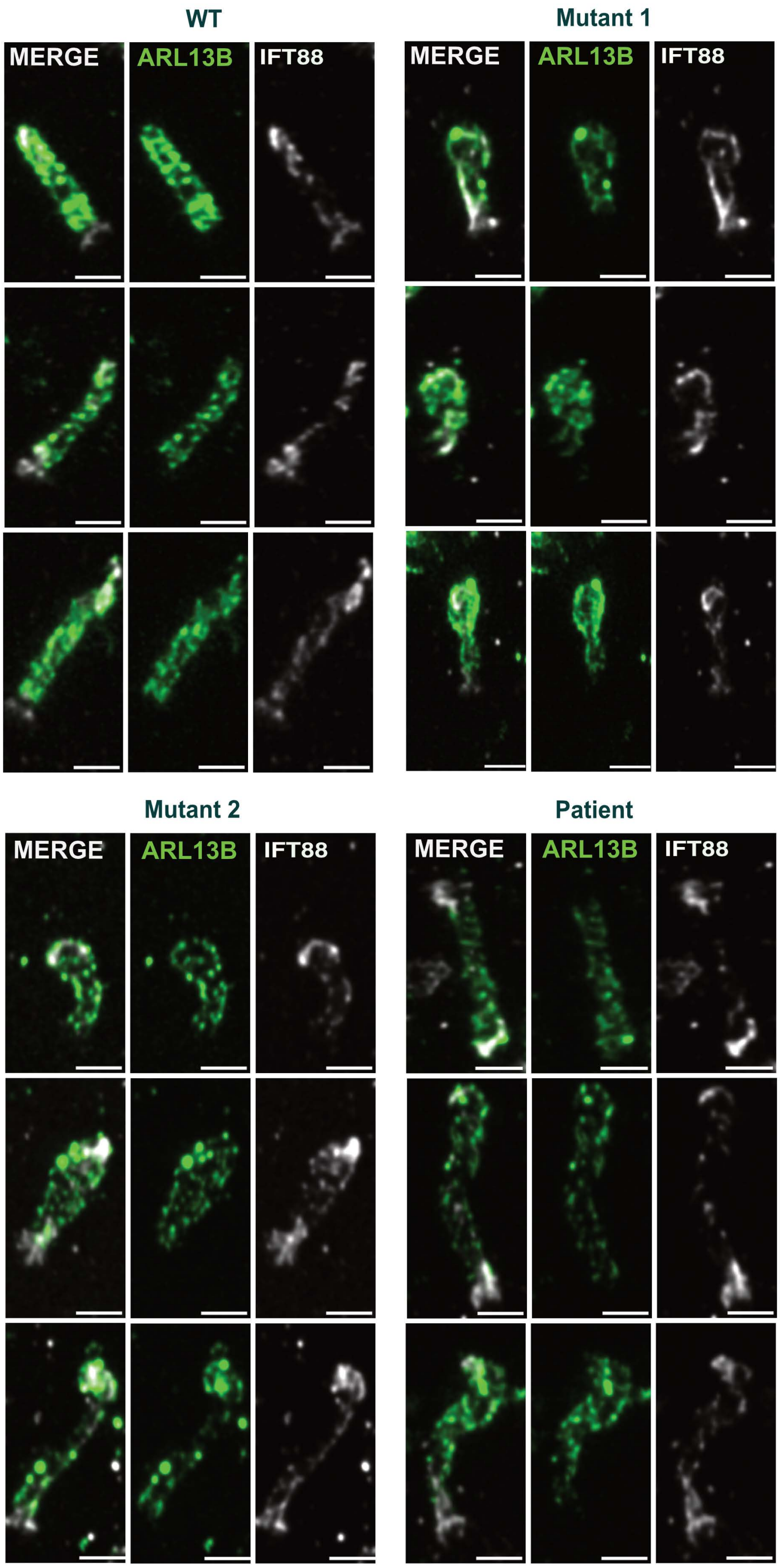

Journal of Cell Science • Supplementary information

Fig. S14. Control and morphologically abnormal *CEP290* mutant ventricular lumen cilia show comparable IFT88 distribution patterns
Immunohistochemistry with expansion super-resolution microscopy (UxEM) of cryosections of 30-day old control (top left), CEP290 mutant 1 (top right), *CEP290* mutant 2 (bottom left) and patient-hiPSC clone C3-derived brain organoids (bottom right) stained with ciliary membrane marker ARL13B (green) and IFT88 (white). IFT88 does not appear to be accumulating abnormally in expanded ventricular cilia of *CEP290* Mutant and CEP290 Patient derived brain organoids. Scale bars (postexpansion physical scale): 2.1 µm (corresponds to 500 nm, corrected for 4.2 expansion factor).

Journal of Cell Science • Supplementary information

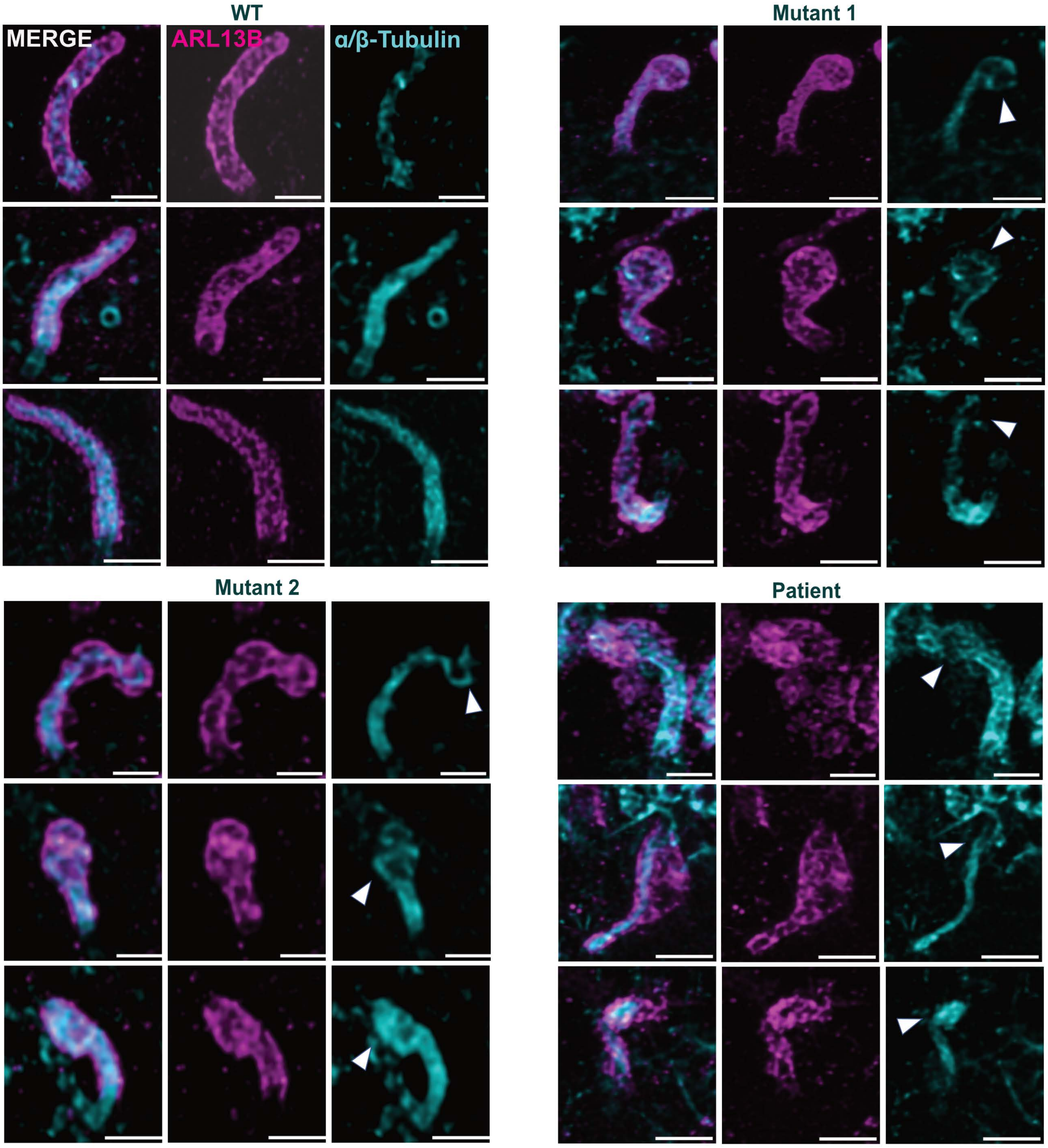

Fig. S15. Gallery of ciliary phenotypes observed in *CEP290* mutant organoids

Immunohistochemistry with expansion super-resolution microscopy (UxEM) of cryosections of 30-day old control (top left) *CEP290* mutant 1 (top right), *CEP290* mutant 2 (bottom left) and patient-hiPSC clone C3-derived brain organoids (bottom right) stained with ciliary membrane marker ARL13B (purple) and α/β-tubulin (cyan). The arrowheads point to splayed microtubules in expanded ciliary tips. Scale bars (post-expansion physical scale): 2.1 µm (corresponds to 500 nm, corrected for 4.2 expansion factor).

Table S1. Quantification of organoid sizes (raw data file for graph in Fig. S8)

Available for download at
https://journals.biologists.com/jcs/article-lookup/doi/10.1242/jcs.264092#supplementary-data

Table S2. Wnt signaling: Differential expression analysis between

- mutant cells from the choroid plexus cluster (cluster 7) and all other mutant cells, for Wnt pathway genes according to KEGG Wnt signaling pathway (hsa04310) (Tab "*Wnt cluster 7 vs other clusters"*)
- mutant cells and control cells from cluster 7 (choroid plexus), for Wnt pathway genes according to KEGG that are significantly more highly expressed in cluster 7 compared to other clusters (Tab "*cl 7 mut vs co high expression"*)
- mutant cells and control cells from cluster 7 (choroid plexus), for additional manually selected Wnt pathway genes (Tab "*cl 7 mut vs co selected Wnt*")
- mutant cells and control cells from cluster 7 (choroid plexus), for downstream targets of Wnt pathway signaling according to the WNT homepage from the Nusse lab at Stanford university https://wnt.stanford.edu/target_genes) (Tab "*cl 7 mut vs co Wnt target genes*").

Available for download at
https://journals.biologists.com/jcs/article-lookup/doi/10.1242/jcs.264092#supplementary-data

Journal of Cell Science • Supplementary information

Table S3. Hedgehog signaling: Differential expression analysis of selected canonical hedgehog signaling genes between

- mutant cells from the choroid plexus cluster (cluster 7) and all other mutant cells (Tab "*HH cluster 7 compared to others*")
- mutant and control cells in the choroid plexus cluster (Tab "*comparison mut vs co cluster 7*")

Available for download at
https://journals.biologists.com/jcs/article-lookup/doi/10.1242/jcs.264092#supplementary-data

Table S4. Differential expression analysis between mutant and wildtype cells

Available for download at
https://journals.biologists.com/jcs/article-lookup/doi/10.1242/jcs.264092#supplementary-data

Table S5. Gene set enrichment analysis on fold changes from DE analysis between mutant and wildtype cells

Available for download at
https://journals.biologists.com/jcs/article-lookup/doi/10.1242/jcs.264092#supplementary-data

Journal of Cell Science • Supplementary information

Table S6. Selected ciliary genes: Differential expression analysis of selected ciliary genes (including genes from various ciliary subcompartments and known CEP290 interactors) between

- mutant cells from the choroid plexus cluster (cluster 7) and all other mutant cells from all other clusters (Tab "*ciliary genes cluster 7 vs rest*")
- mutant and control cells in the choroid plexus cluster 7 (Tab "*mutant vs control cluster in 7*")

- all mutant and control cells (Tab "*mutant vs control all cells*")

Available for download at
https://journals.biologists.com/jcs/article-lookup/doi/10.1242/jcs.264092#supplementary-data

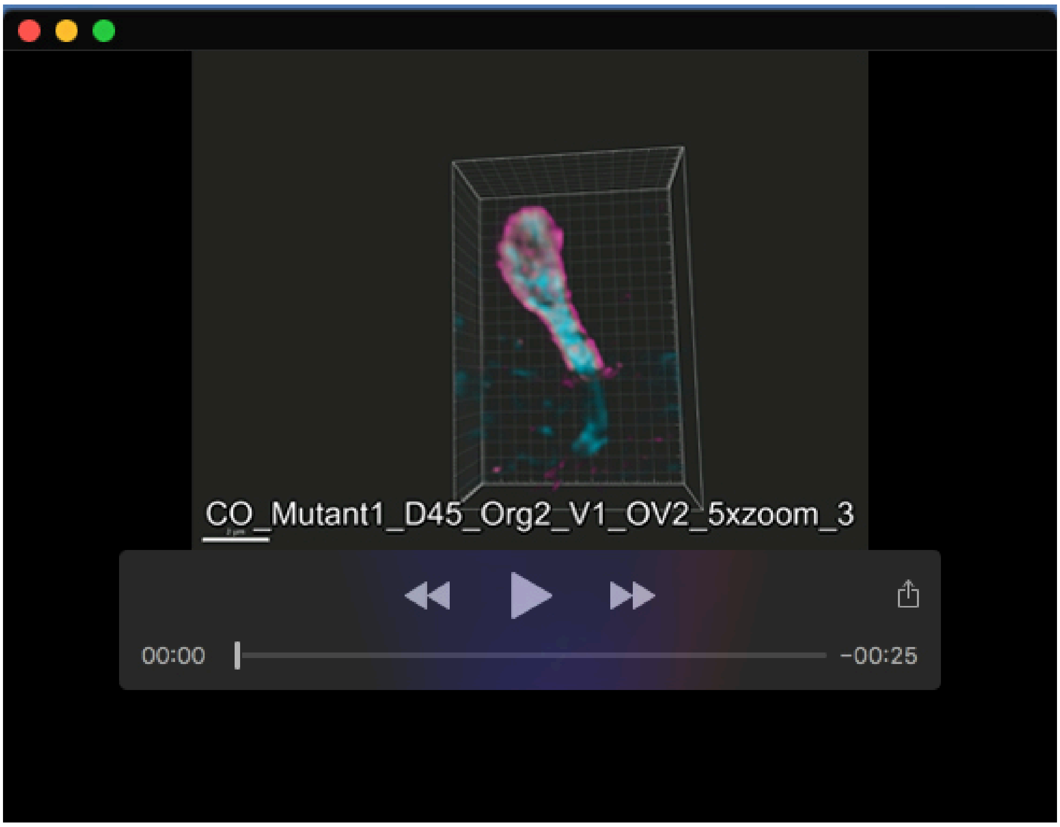

Movie 1. 3D visualization of dysmorphic cilia after expansion microscopy. Related to main Fig. 3 and Fig. S14.

Journal of Cell Science • Supplementary information
